# Loss of pH Control in *Plasmodium falciparum* Parasites Subjected to Oxidative Stress

**DOI:** 10.1371/journal.pone.0058933

**Published:** 2013-03-11

**Authors:** Donelly A. van Schalkwyk, Kevin J. Saliba, Giancarlo A. Biagini, Patrick G. Bray, Kiaran Kirk

**Affiliations:** 1 Research School of Biology, The Australian National University, Canberra, Australian Capital Territory, Australia; 2 Medical School, The Australian National University, Canberra, Australian Capital Territory, Australia; 3 Liverpool School of Tropical Medicine, Liverpool, United Kingdom; Univ. Georgia, United States of America

## Abstract

The intraerythrocytic malaria parasite is susceptible to oxidative stress and this may play a role in the mechanism of action of some antimalarial agents. Here we show that exposure of the intraerythrocytic malaria parasite to the oxidising agent hydrogen peroxide results in a fall in the intracellular ATP level and inhibition of the parasite's V-type H^+^-ATPase, causing a loss of pH control in both the parasite cytosol and the internal digestive vacuole. In contrast to the V-type H^+^-ATPase, the parasite's digestive vacuole H^+^-pyrophosphatase is insensitive to hydrogen peroxide-induced oxidative stress. This work provides insights into the effects of oxidative stress on the intraerythrocytic parasite, as well as providing an alternative possible explanation for a previous report that light-induced oxidative stress causes selective lysis of the parasite's digestive vacuole.

## Introduction

As it grows within its host erythrocyte, the unicellular malaria parasite is exposed to oxidative stress [1]. Haemoglobin in the host erythrocyte cytosol undergoes autoxidation to methaemoglobin, resulting in the generation of reactive oxygen radicals (O_2_^−^) [2]. Furthermore, the digestion of haemoglobin within the acidic digestive vacuole (DV) of the parasite, increases the conversion of oxyhaemoglobin to methaemoglobin and the accompanying release of hydrogen peroxide (H_2_O_2_) [3]. In the *in vivo* situation, the parasite is also exposed to products of the oxidative burst of macrophages activated by the host immune system during malarial infection [4], [5].

The parasite has a range of antioxidant defence mechanisms. It uses the reducing activity of two thiol-containing compounds, glutathione and thioredoxin, to combat oxidative agents such as H_2_O_2_ and superoxide (reviewed in [6]). Although lacking the enzymes catalase and glutathione peroxidase, the parasite encodes a range of peroxiredoxins, which are used to detoxify oxygen radicals [7], and there is also evidence that the intraerythrocytic parasite imports the human peroxiredoxin 2 from the host cell for the purpose of detoxifying peroxides [8].

Despite having such antioxidant mechanisms, the parasite is susceptible to oxidative damage. Several studies have demonstrated the sensitivity of intraerythrocytic parasite growth, *in vivo* or *in vitro*, to exogenous oxidising agents such as H_2_O_2_ [4], [5], [9], alloxan [10], divicine [11] and tert-butyl hydroperoxide (t-BOOH) [12]. There is evidence that artemisinin, now a mainstay of antimalarial chemotherapy, exerts its antimalarial activity, at least in part, through the generation of oxidative stress [13]–[14], and parasites showing reduced susceptibility to artemisinin following prolonged drug exposure show elevation of the oxidative defence network [15]. Oxidative stress may also be a component of the mechanism of action of other antimalarial agents (e.g. [16], [17]). However, there is little information on specific targets within the parasite that result in this susceptibility to oxidative stress. H_2_O_2_ or t-BOOH are known to cause lipid peroxidation in both uninfected and parasitised erythrocytes [12], [18]. Wissing *et al*. have reported that lipid peroxidation resulting from light-induced generation of hydroxyl radicals causes selective disruption of the membrane of the parasite's acidic digestive vacuole (DV) [19] and Radfar *et al.* (2008) have identified a range of *P. falciparum* encoded proteins that undergo oxidative damage in response to chloroquine treatment of parasitised erythrocytes [16].

In this study, we have investigated the effect of the oxidising agent H_2_O_2_ on aspects of the biochemistry of the intraerythrocytic malaria parasite. Addition of H_2_O_2_ to parasites resulted in an acidification of the parasite cytosol and alkalinisation of its digestive vacuole, as well as causing a decrease in parasite ATP levels. The oxidising agent was shown to inhibit the parasite's DV H^+^-pumping V-type H^+^-ATPase directly, while having no effect on the activity of the DV H^+^-pumping pyrophosphatase. The oxidising agent therefore disrupts pH regulation in the parasite both by direct inhibition of the V-type H^+^-ATPase, and, indirectly, by reducing the intracellular ATP concentration, thereby depriving the V-type H^+^-ATPase of the fuel required to pump H^+^ ions both into the DV and out of the parasite, across the parasite plasma membrane.

## Materials and Methods

### Materials

Hydrogen peroxide (H_2_O_2_), firefly lantern extract and nigericin were purchased from Sigma Chemical Co. (St. Louis, MO, USA). Concanamycin A was purchased from MP Biomedicals (Santa Ana, CA, USA). Albumax II, gentamicin sulphate, HEPES, fluorescein-dextran (*M*_r_ 10000), SNARF-1 and the acetoxymethyl ester form of the fluorescent pH indicator 2',7'-bis-(2-carboxyethyl)-5-(and-6)-carboxyfluorescein (BCECF) were purchased from Invitrogen (Carlsbad, CA, USA).

### Cell preparations

Experiments were performed on the 3D7 or D10 strains of *Plasmodium falciparum* or, in one series of experiments, on transfectant Dd2 parasites expressing pH-sensitive chimeras of green fluorescent protein (GFP) with the DV haemoglobinase plasmepsin II (PM2) [20]. The parasites were maintained at 37 °C in O^+^ human erythrocytes suspended, by continuous shaking, in RPMI-1640 culture medium as described previously [21]. The culture medium was supplemented with sodium bicarbonate (25 mM), gentamicin sulphate (24 µg/mL), glucose (11 mM), HEPES (25 mM), hypoxanthine (200 µM) and Albumax II (6 g/L) and the suspension was maintained under a gas mixture of 3% CO_2_, 1% O_2_ and 96% N_2_. Cultures were synchronized at the ring stage by dilution in 10 volumes of 5% (w/v) D-sorbitol as described elsewhere [22].

The majority of experiments were carried out with mature, trophozoite-stage parasites (36–40 hours post-invasion) functionally isolated from their host cells by permeabilisation of the erythrocyte and parasitophorous vacuole membranes using saponin (0.05% w/v, yielding a 0.005% w/v concentration of the active agent sapogenin) as described elsewhere [23], [24].

In one series of experiments, measurements were carried out using a preparation in which the plasma membrane of saponin-isolated parasites was permeabilised with digitonin (0.01% w/v), as described elsewhere [23]. Permeabilisation of the parasite plasma membrane allows solutes (such as ATP and inorganic pyrophosphate (PP_i_)) added to the extracellular solution to gain access to the surface of the DV.

### Measurement of pH_i_ and pH_DV_ in cell populations

The effect of oxidising agents on the parasite's cytosolic pH (pH_i_) was investigated in suspensions of isolated parasites preloaded with the pH-sensitive fluorescent dye BCECF, as described previously [23]. The BCECF-loaded parasites were suspended in HEPES-buffered saline (120 mM NaCl, 5 mM KCl, 25 mM HEPES, 20 mM glucose and 1 mM MgCl_2_, pH 7.1) at a density of approximately 5×10^7^ cells/mL. Fluorescence measurements were made (at 37°C) by exciting the suspension at both 440 nm and 495 nm and recording the fluorescence at an emission wavelength of 520 nm, using either a Perkin-Elmer LS 50B fluorometer (PerkinElmer Life and Analytical Sciences, Waltham, MA, USA) with a “Fast Filter” accessory, or a FLUOstar Optima microplate reader (BMG Labtech, Durham, NC, USA). The ratio of the fluorescence measured at the dual wavelengths (490 nm/440 nm) provided a measure of the pH, with calibration carried out as described elsewhere [25].

The pH of the parasite's digestive vacuole (pH_DV_) was monitored in parasites in which the DV had been loaded with the pH sensitive dye, fluorescein-dextran, as described elsewhere [23], [26], or in transfectant parasites expressing the pH-sensitive GFP-PM2 fusion protein in the DV [20], as described by Lehane *et al.* [27]. The measurements were carried out in the fluorometer on suspensions of saponin-isolated parasites at 37°C. The fluorescence emanating from the transfectant parasites was much lower than that from the fluorescein-dextran-loaded parasites and it was therefore necessary to use a much higher concentration of cells in the fluorometer cuvette in the experiments with the transfectant parasites (7×10^7^ cells/ml) than in those with the dye-loaded parasites (∼5×10^6^ cells/ml).

### Single cell measurement of pH_i_ and pH_DV_

Single cell estimates of pH_i_ and pH_DV_ of parasites functionally isolated from their host cells by saponin-permeabilisation of the erythrocyte membrane (as described above) were made using confocal microscopy. pH_i_ estimates were made in parasites loaded with SNARF by incubation of the isolated parasites with SNARF-AM (5 µM) for 10 min at 37°C. Estimates of pH_DV_ were made in parasites in which the DV had been preloaded with fluorescein-dextran as described elsewhere [23], [26]. The isolated parasites were washed by centrifugation (1800×g, 5 min), resuspended in HEPES-buffered saline, then immobilized on polylysine coated coverslips in a Bioptechs FCS2 perfusion chamber at 22 °C. The fluorescence signals from the isolated parasites were collected on a Zeiss Pascal confocal laser scanning microscope through a Plan-Apochromat 63×1.2 numerical aperture water objective. The fluorescein-dextran fluorescence was excited at 458 nm and collected off a 545 nm dichroic mirror through a 500–530 nm band pass filter. The SNARF fluorescence was excited at 543 nm and collected through a 545 nm dichroic mirror and 560 nm long-pass filter. Photobleaching was assessed by continuous exposure (5 min) of loaded cells to laser illumination. Data capture and extraction were carried out with Zeiss physiology imaging software.

### ATP Measurements

The concentration of ATP in isolated parasites was measured using firefly luciferase as described elsewhere [28]. Briefly, saponin-isolated parasites were suspended (at a final cell density of 4–5×10^7^ cells/mL) in HEPES-buffered saline and dispensed in 50 µL aliquots into a 96-well microtitre plate in the absence or presence of H_2_O_2_. At selected time points, ATP synthesis/utilization was terminated by the addition of 100 µL of HCl (0.1 M), after which, 75 µL of the solution was removed and diluted in 200 µL of water. Aliquots (15 µL) of the resulting solution were dispensed into the wells of a white 96-well plate (opaque plates were used to eliminate ‘cross-talk’ between wells). A buffered solution (185 µL) composed of 20 mM HEPES, 25 mM MgCl_2_, 5 mM Na_2_HPO_4_, and firefly lantern extract (1% v/v), pH 7.4, was added to each well. The luminescence was measured immediately in a FLUOstar Optima microplate reader (BMG Labtech, Durham, NC). An ATP calibration curve (0.04–4.5 µM; linear over this range) was performed in each experiment and used to estimate the intracellular ATP concentration, assuming a parasite volume of 28 fL [29].

### Statistics

All statistical comparisons were made using a paired two-tailed Student's t-test.

## Results

### H_2_O_2_ acidifies the cytosol and decreases [ATP] in isolated parasites

**Figure 1:**
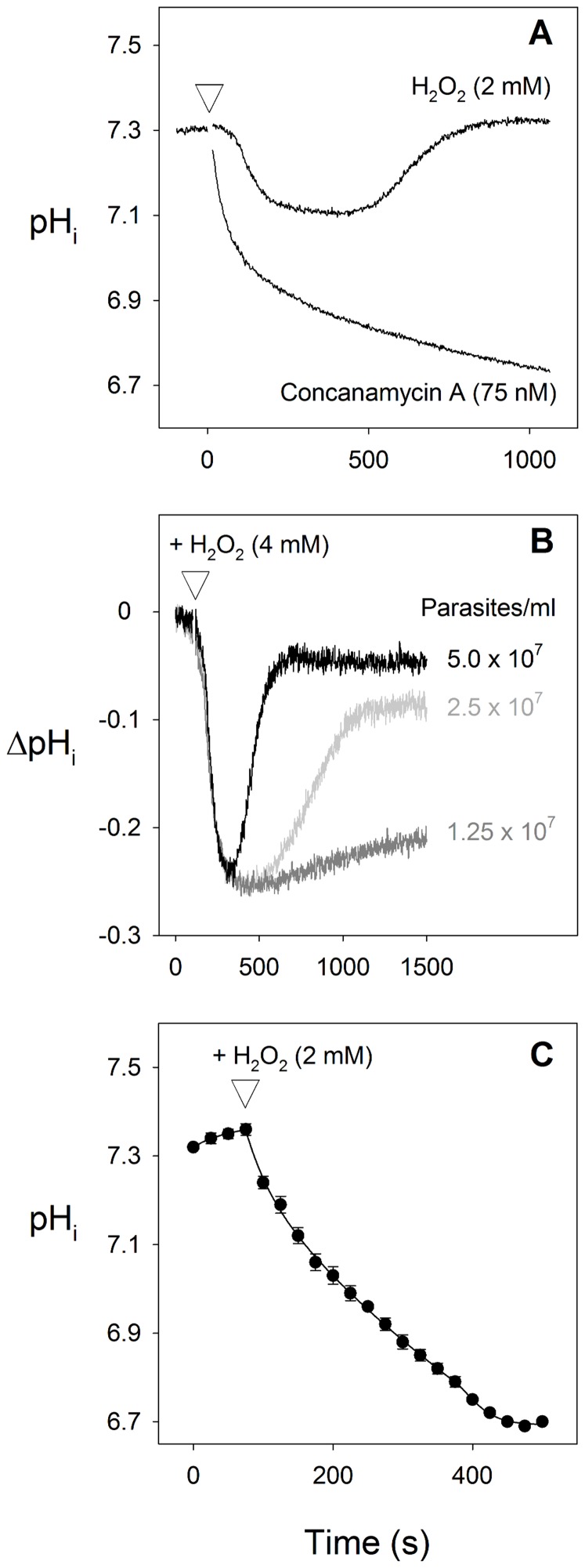
Effect of the oxidising agent H_2_O_2_ on pH_i_ in isolated parasites. (**A**) Effect of H_2_O_2_ (2 mM) and the V-type H^+^ ATPase inhibitor concanamycin A (75 nM) on pH_i_. The reagents were added at the point indicated by the white triangle. The traces shown are from a single experiment on a suspension of isolated BCECF-loaded 3D7 *P. falciparum* trophozoites (5×10^7^ cells/ml) at 37°C and are representative of those obtained in five similar experiments. (**B**) Effect of parasite concentration on the response of pH_i_ to the oxidising agent H_2_O_2_ (4 mM, added at the point indicated by the white triangle) in suspensions of isolated BCECF-loaded 3D7 parasites at 37°C. ΔpH_i_ indicates the deviation from the initial resting pH_i_. The traces shown are from a single experiment but are representative of those obtained in three similar experiments. (**C**) Effect of H_2_O_2_ (2 mM, added at the point indicated by the white triangle) on pH_i_ in single isolated SNARF-loaded D10 parasites immobilized on polylysine coated coverslips at 22°C. The data showing cytosolic pH_i_ are averaged from 79 individual cells carried out on three different days, and are shown±S.D.

As shown in Fig. 1A, addition of 2 mM H_2_O_2_ to suspensions of BCECF-loaded isolated parasites at 37°C caused a transient acidification of the parasite cytosol. The transient nature of the pH_i_-response to H_2_O_2_ contrasted with the sustained acidification of the cytosol seen following the addition of the V-type H^+^-ATPase inhibitor concanamycin A (75 nM; Fig. 1A).

In replicate experiments the average resting cytosolic pH prior to the addition of H_2_O_2_ or concanamycin A was 7.35±0.03 (mean±SEM, n = 8). By 500 s after the addition of 2 mM H_2_O_2_ the average pH_i_ was reduced significantly from the resting value, to 7.16±0.05 (P = 0.007, n = 8). By 1000 s after the H_2_O_2_ addition pH_i_ had recovered to 7.31±0.03, not significantly different from the original resting pH_i_ (P = 0.234, n = 8). In the case of the concanamycin A-treated cells the average pH_i_ 500 s after the addition of 75 nM concanamycin A was reduced significantly from the resting value, to 6.94±0.03 (P = 0.0001, n = 6). By 1000 s after the concanamycin A addition the average pH_i_ had decreased further to 6.86±0.02, again significantly lower than the original resting pH_i_ (P<0.0001, n = 8).

As is illustrated in Fig. 1B, the duration (but not the magnitude) of the cytosolic acidification induced by the addition of H_2_O_2_ varied with the concentration of parasites in the suspension, increasing as the concentration of parasites was decreased.

In single cell studies, in which the cytosolic pH was monitored in single isolated parasites immobilized on polylysine coated coverslips at 22°C, 2 mM H_2_O_2_ again caused a cytosolic acidification which, in this case, did not reverse over the 500 s timeframe of the experiment (Fig. 1C). These single cell studies use a much lower cell concentration, as well as a lower temperature, than was used in the cell suspension studies giving rise to Figs 1A and 1B. The observation that the parasites failed to recover from the H_2_O_2_-induced cytosolic acidification over the time course of the experiment is therefore in keeping with the data shown in Fig. 1B, as well as being likely to be due, in part, to the lower temperature.

The dependence of the duration of the H_2_O_2_-induced acidification on cell concentration is consistent with the parasites themselves inactivating the oxidising agent. This was confirmed in experiments in which H_2_O_2_ (2 mM) was preincubated (for 15 min at 37 °C) either in the presence or absence of isolated (intact) parasites (5×10^7^ cells/ml) before being added to a suspension of dye-loaded parasites and pH_i_ monitored. Addition to parasites of H_2_O_2_ solution that had been preincubated in the absence of parasites caused a transient decrease in pH_i_ (as in Fig. 1A and B), whereas addition of H_2_O_2_ solution that had been preincubated with parasites had no effect on pH_i_ (data not shown). The transient nature of the parasite's pH_i_ response is therefore attributable to the parasite's ability to inactivate H_2_O_2_ via its anti-oxidant defence mechanisms. Whether the inactivation of H_2_O_2_ by the parasite occurred within the parasite, via the parasite's internal antioxidant defence mechanisms, or in the extracellular medium (perhaps involving exported glutathione; [30]), was not investigated.

**Figure 2:**
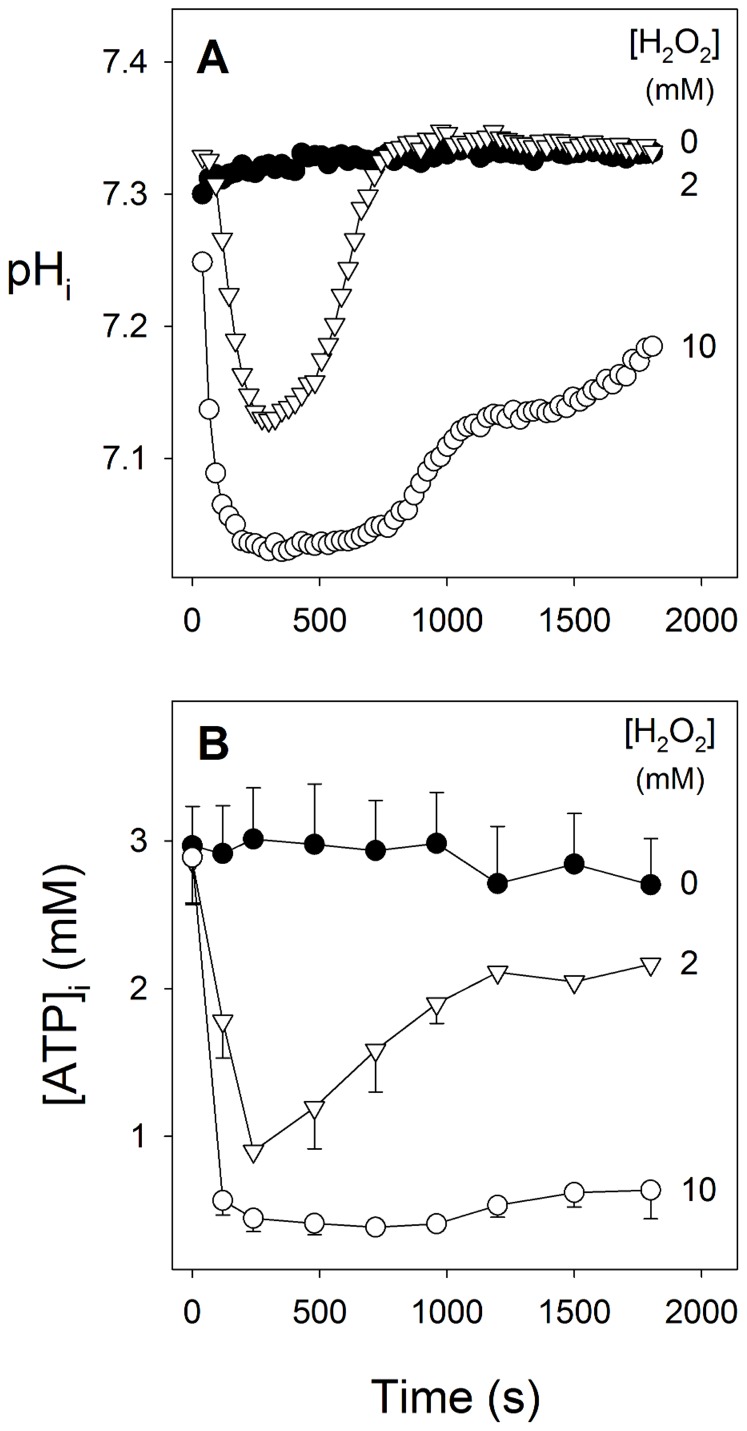
Concentration-dependent effects of H_2_O_2_ on (A) cytosolic pH (pH_i_) and (B) [ATP]_I_ in isolated parasites. Isolated 3D7 *P. falciparum* trophozoites were loaded with the pH-sensitive fluorescent dye BCECF. H_2_O_2_ was either absent (black circles) or added at time-zero at a concentration of either 2 mM (triangles) or 10 mM (open circles). The data are averaged from three separate experiments. In (A) the error bars were omitted for clarity; in the control experiment the S.E.M. ranged from 0.003–0.013 pH units, in the 2 mM H_2_O_2_ experiment the S.E.M. ranged from 0.003–0.092 pH units, and in the 10 mM H_2_O_2_ experiment the S.E.M. ranged from 0.008–0.109 pH units. In (B) the error bars denote S.E.M.

As illustrated in Fig. 2A, the effect of H_2_O_2_ on cytosolic pH was concentration-dependent. In these experiments (n = 4) the addition of 2 mM H_2_O_2_ caused an average maximum decrease in pH_i_ of 0.19±0.05 units. The average maximum decrease seen on addition of 10 mM H_2_O_2_, 0.29±0.02 pH units, was slightly larger, though not significantly so (P = 0.10). In parasites acidified by exposure to 2 mM H_2_O_2_ pH_i_ recovered to its original level within 10–15 min. By contrast, after approximately 10 min of exposure of parasites to the higher (10 mM) H_2_O_2_ concentration pH_i_ did begin to recover, but recovery was still incomplete within the 3 min timeframe of the experiment.

The maintenance of pH_i_ in the intraerythrocytic parasite is reliant on a plasma membrane V-type H^+^-ATPase [24], [25], [31] and, therefore, on intracellular ATP. As shown in Fig. 2B, addition of H_2_O_2_ to isolated parasites caused a concentration-dependent, transient reduction in the intracellular ATP concentration ([ATP]_i_). Addition of 2 mM H_2_O_2_ caused [ATP]_i_ in the isolated parasite to decrease from an initial average resting value of 2.97±0.30 mM to an average value of 0.90±0.06 mM at 240 s after the addition, significantly lower than the value measured at the same time in cells incubated under control conditions (3.01±0.35 mM, P = 0.02, n = 3). By 1800 s after the addition of 2 mM H_2_O_2_ [ATP]_i_ had recovered to an average value of 2.17±0.11 mM, not significantly different from the value of 2.70±0.31 mM measured in cells incubated for the same period under control conditions (P = 0.13, n = 3). On addition of 10 mM H_2_O_2_, [ATP]_i_ decreased to an average value of 0.45±0.09 mM at 240 s after the addition, significantly lower than the value measured at the same time in cells incubated under control conditions (P = 0.01, n = 3). By 1800 s after the addition of 10 mM H_2_O_2_ [ATP]_i_ had recovered slightly, to an average value of 0.64±0.19 mM, but was still significantly lower than that measured in cells incubated for the same period under control conditions (P = 0.0489, n = 3).

In control experiments it was shown that in ATP solutions (in the absence of cells), H_2_O_2_, at concentrations ranging from 2–50 mM, had no effect on the ATP concentration (data not shown). The decrease in [ATP]_i_ seen in isolated parasite suspensions is therefore not attributable to the oxidising agent breaking ATP down via a direct (chemical) interaction, or to an effect on the ATP assay.

### H_2_O_2_ alkalinises the digestive vacuole and inhibits the V-type H^+^-ATPase, but not the H^+^-PPase, in isolated parasites

The pH of the parasite's internal DV (pH_DV_) is maintained in the range 4.9–5.9 [20], [32], [33], [34], primarily through the action of a V-type H^+^-ATPase on the DV membrane [23]. A second H^+^ pump, a H^+^-pumping pyrophosphatase (H^+^-PPase), is also present on this membrane and can acidify the DV (independently of the H^+^-ATPase) in the presence of PP_i_ [23].

**Figure 3:**
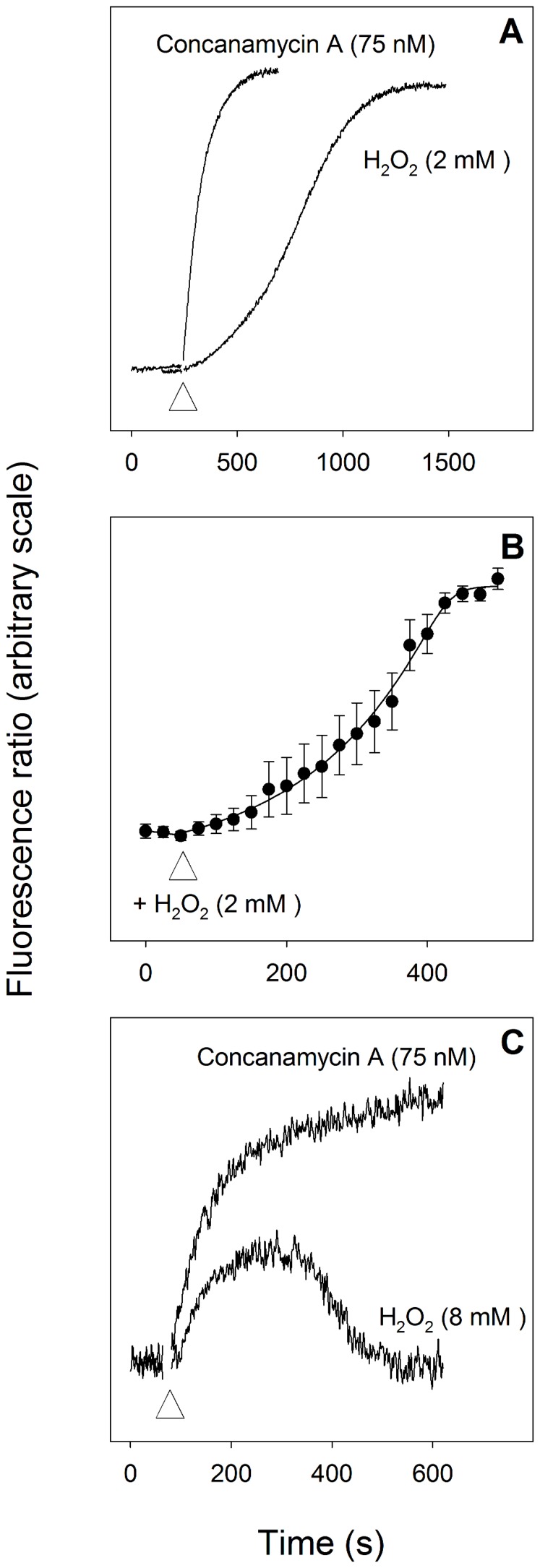
Effect of H_2_O_2_ on pH_DV_ in isolated parasites. (**A**) Effect of H_2_O_2_ (2 mM) and the V-type H^+^ ATPase inhibitor concanamycin A (75 nM) on pH_DV_ in suspensions of isolated D10 trophozoites in which the DV was preloaded with the membrane-impermeant pH-sensitive dye, fluorescein-dextran. The cells were suspended at a density of ∼5×10^6^ cells/ml at 37°C and the reagents were added at the point indicated by the white triangle. The fluorescence measurements were not calibrated; an increase in the fluorescence ratio is indicative of an increase in pH_DV_ (i.e. an alkalinisation). The traces shown are from a single experiment and are representative of those obtained in five similar experiments. (**B**) Single cell measurements showing the effect of H_2_O_2_ (2 mM, added at the point indicated by the white triangle) on pH_DV_ of isolated D10 *P. falciparum* trophozoites in which the DV was preloaded with the membrane-impermeant pH-sensitive dye, fluorescein-dextran. An increase in the fluorescence ratio is indicative of an increase in pH_DV_. The data are averaged from 48 individual parasites carried out at 22°C on three different days, and are shown±S.D. (**C**) Effect of H_2_O_2_ (8 mM) and concanamycin A (75 nM) on pH_DV_ in suspensions of Dd2 transfectant parasites expressing a pH-sensitive GFP-PM2 fusion protein in the DV, and suspended at a density of 7×10^7^ cells/ml at 37°C. The reagents were added at the point indicated by the white triangle. An increase in the fluorescence ratio is indicative of an increase in pH_DV_.

As shown in Fig. 3A, addition of H_2_O_2_ (2 mM) to suspensions of isolated parasites in which the digestive vacuole had been loaded with the membrane-impermeant pH-sensitive dye, fluorescein-dextran, caused an alkalinisation of the DV, with the increase being similar in magnitude to (albeit somewhat slower than) that caused by addition of the V-type H^+^-ATPase inhibitor concanamycin A (75 nM). It should be noted that the cell concentration used in the experiment giving rise to Fig. 3A was some 10-fold lower than the cell concentration used in the experiment giving rise to Fig. 1A, and it is likely for this reason that the H_2_O_2_-induced change in the pH of the DV did not reverse on the timescale of the experiment (Fig. 3A) whereas the H_2_O_2_-induced change in cytosolic pH did (Fig. 1A).

A similar phenomenon was seen both in single cells (Fig. 3B) and in suspensions of transfectant parasites expressing a pH-sensitive GFP-PM2 fusion protein in the DV (Fig. 3C). The addition of H_2_O_2_ (8 mM) to the transfectants resulted in an immediate alkalinisation of the DV. There were quantitative differences between the results obtained with fluorescein-dextran-loaded parasites (Fig. 3A) and transfectant parasites (Fig. 3C). The onset of the increase in pH_DV_ seen following the addition of 8 mM H_2_O_2_ to the transfectant parasites (Fig. 3B) was faster than that seen in response to the addition of 2 mM H_2_O_2_ to fluorescein-dextran-loaded parasites (Fig. 3A). Furthermore, in contrast to the results obtained with dye-loaded parasites, the magnitude of the H_2_O_2_-induced alkalinisation of the transfectant parasites was less than that seen in response to the addition of concanamycin A, and was transient, with pH_DV_ returning to its initial starting value within 10 min. Addition of lower concentrations of H_2_O_2_ (2–4 mM) to the transfectant parasites gave rise to transient DV alkalinisations that were slower, smaller and shorter (not shown).

The finding that the size and duration of the H_2_O_2_-induced alkalinisation seen in the transfectant parasites (Fig. 3C) were less than those seen in fluorescein-dextran-loaded parasites (Fig. 3A and B), as well as being transient (whereas that in fluorescein-dextran-loaded parasites was not), may be accounted for by the fact that the lower fluorescence signal from the transfectant parasites necessitated the use of a higher concentration of these cells in order to gain a sufficient fluorescence signal; in the experiment giving rise to Fig. 3A the cell concentration was ∼5×10^6^ cells/ml whereas in that giving rise to Fig. 3C the concentration was 7×10^7^ cells/ml (i.e. some 14-fold higher). The higher density cell suspension used for the transfectants would be expected to inactivate the H_2_O_2_ (resulting in a reversal of the DV alkalinisation) much more rapidly than the lower density suspension of dye-loaded cells.

**Figure 4:**
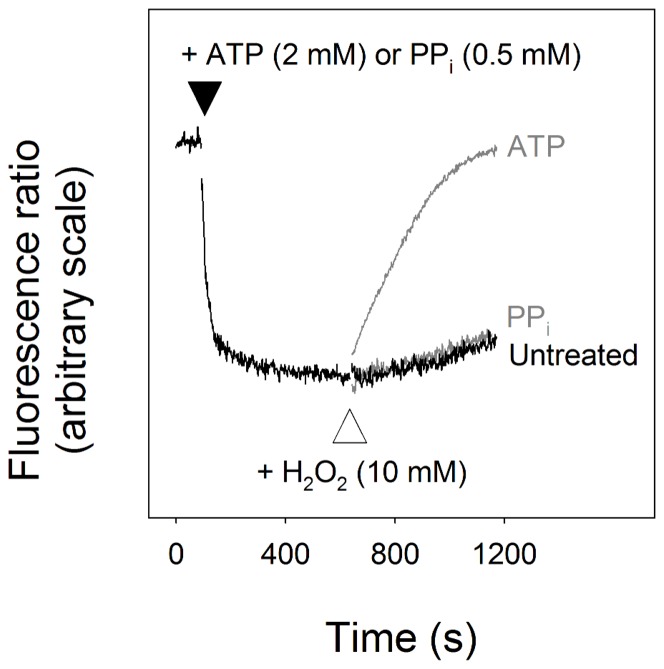
Effect of H_2_O_2_ on the ability of the parasite to maintain an acidic DV. Digitonin-permeabilised 3D7 trophozoites in which the DV was preloaded with fluorescein-dextran were suspended at a density of ∼5×10^6^ cells/ml at 37°C. The fluorescence measurements were not calibrated; an increase in the fluorescence ratio is indicative of an increase in pH_DV_. The addition of either 2 mM ATP or 0.5 mM PPi to the external medium at the point indicated by the black triangle caused a rapid acidification of the vacuole (the trace shown is that obtained following the addition of PP_i_; a very similar trace was observed on addition of ATP). On addition of H_2_O_2_ (10 mM, at the point indicated by the white triangle) to the permeabilised parasites (in the continued presence of ATP or PP_i_) there was an immediate alkalinisation in those parasites in which the DV was acidified by the addition of ATP (light grey trace), whereas in those parasites in which the DV was acidified by the addition of PP_i_ the pH_DV_ was largely unaffected (dark grey trace). These data are consistent with H_2_O_2_ inhibiting the parasite's V-type H^+^-ATPase while not inhibiting the H^+^-PPase. The traces shown are from a single experiment and are representative of results obtained from at least three similar experiments.

As illustrated in Fig. 2B, H_2_O_2_ caused a decrease in parasite ATP levels. This, of itself, might be predicted to result in alkalinisation of the DV (by depriving the DV H^+^-ATPase of its substrate). The question of whether H_2_O_2_ inhibits DV H^+^ pumps directly (i.e. independently of the effect on [ATP]_i_) was addressed in parasites in which the plasma membrane was permeabilised by the addition of digitonin, allowing substrates added to the external medium to gain access to the DV membrane. As shown in Fig. 4, addition of either ATP (2 mM) or PP_i_ (0.5 mM) to permeabilised parasites, resulted in a rapid acidification of the DV. The acidification seen in response to the addition of ATP is attributable to the DV H^+^-ATPase whereas the alkalinisation seen in response to the addition of PP_i_ is attributable to the DV H^+^-PPase. In the case of parasites in which the DV had been acidified by the action of the H^+^-ATPase (following the addition of ATP), addition of H_2_O_2_ (10 mM) caused a rapid alkalinisation, consistent with the oxidising agent causing a direct inhibition of the V-type H^+^-ATPase. By contrast, in permeabilised parasites that had been acidified via the action of the H^+^-PPase (following the addition of PP_i_), addition of the same concentration of H_2_O_2_ (10 mM) had no significant effect, consistent with the H^+^-PPase being refractory to inhibition by the oxidising agent. The observation that on addition of H_2_O_2_ to permeabilised parasites in which the DV was acidified by the addition of the PPi there was no increase in fluorescence (relative to the control) indicates that the DV remains intact and retains the fluorescent indicator in the presence of the oxidising agent, ruling out the possibility that the increase in fluorescence seen in the ATP-treated cells was due to the H_2_O_2_ inducing a release of the indicator from the DV.

### H_2_O_2_ does not impair DV membrane integrity

In a previous study of pH regulation in the intraerythrocytic malaria parasite, using confocal microscopy, Wissing *et al.* (2002) reported that in parasites stained with the membrane-permeant pH indicator, acridine orange, the fluorescence pattern changed in response to prolonged illumination with the microscope's laser. Initially acridine-orange-stained cells showed bright red fluorescence associated with the parasite's digestive vacuole, and green fluorescence with the parasite cytosol. However, on prolonged exposure to laser light there was a loss of the red fluorescence from the digestive vacuole compartment. Wissing *et al.* went on to demonstrate a similar laser-light-induced redistribution of another membrane-permeant pH marker, lysosomal blue, and showed that the redistribution of these membrane-permeant fluorescent indicators was due to an acidification of the parasite cytosol. This acidification was attributed to the selective rupture of the parasite's DV as a result of oxidative stress arising from light-induced generation of hydroxyl radicals [19].

In the present study, the ability of pH_DV_ to recover following an H_2_O_2_-induced alkalinisation (Fig. 3C), and the ability of the DV to maintain an acidic pH in the presence of PP_i_, following the addition of 10 mM H_2_O_2_ (Fig. 4) both indicate that the DV remained intact under the conditions of oxidative stress investigated here. Nevertheless, we investigated the integrity of the DV membrane in parasites exposed to very high concentrations of H_2_O_2_, and compared the response of the parasite to H_2_O_2_ with the response of the parasite to laser light.

**Figure 5:**
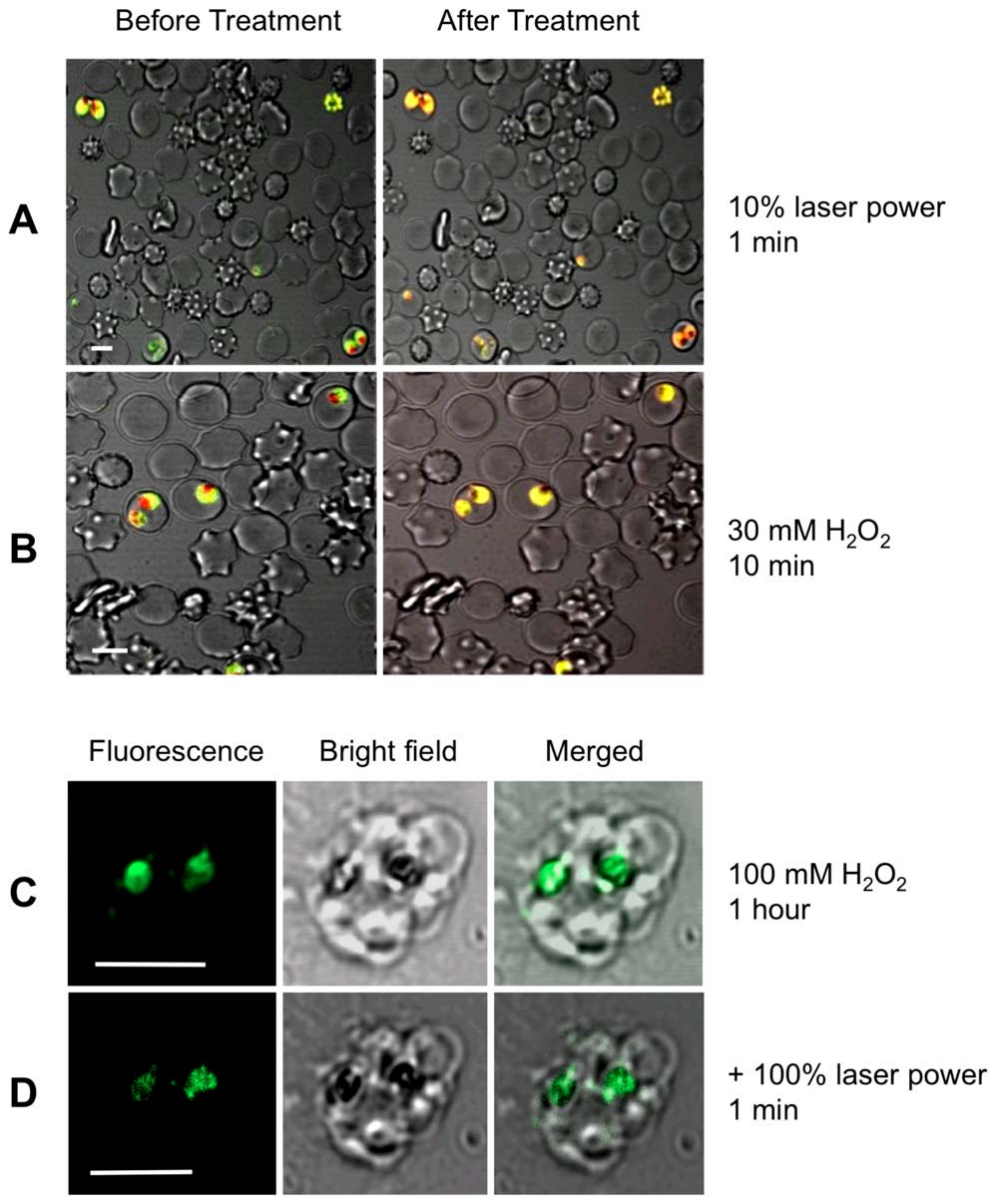
The parasite DV remains intact in parasites subjected to oxidative stress. (**A**) and (**B**) are confocal micrographs showing the redistribution of acridine orange fluorescence in intact parasitized erythrocytes subjected either to (**A**) 1 min illumination with the microscope's laser, or (**B**) 10 min exposure to 30 mM H_2_O_2_. Prior to the treatment the DV fluoresces red and the parasite cytosol fluoresces green. Both treatments resulted in a loss of red fluorescence from the region of the DV. (**C**) and (**D**) show the retention of fluorescein-dextran within the DV of mature, isolated D10 trophozoites (there are two visible in the image) following exposure to 100 mM H_2_O_2_ for 1 hour (**C**), followed by excitation with a 488 nm laser line at full power for 1 min (**D**). Neither the high concentration of H_2_O_2_ alone, nor the subsequent additional intense light exposure resulted in any redistribution of the fluorescein-dextran from the DV of the parasites (visible as dark regions, coinciding with the hemozoin crystals, in the bright-field images), from which it may be concluded that the DV remained intact. All scale bars (shown in white) are 5 µm.

As shown in Fig. 5A, prolonged exposure of acridine orange-stained parasitised erythrocytes to laser light resulted in a redistribution of fluorescence very similar to that described by Wissing *et al.* (2002), with a loss of red fluorescence from the region of the DV. A very similar redistribution of fluorescence was seen on exposure of parasitised erythrocytes to 30 mM H_2_O_2_ (Fig. 5B). The question of whether this redistribution might be attributed to a loss of DV membrane integrity was investigated in experiments in which the parasite DV was loaded with the membrane impermeant fluorescent indicator fluorescein-dextran.

Fluorescein-dextran loaded cells were isolated from their host cells by saponin-permeabilisation of the erythrocyte membrane, fixed to a coverslip, then exposed first to a high concentration of H_2_O_2_ (100 mM) for 1 hour (Fig. 5C), then to the full laser power of the microscope for 1 minute (Fig. 5D). Neither the high concentration of H_2_O_2_ alone, nor the subsequent additional intense light exposure resulted in any redistribution of the fluorescein-dextran from the DV of the parasites; in both cases the fluorescence remained confined to the area corresponding to the hemozoin crystals (as observed in bright field images), consistent with the DV membrane remaining intact.

## Discussion

Although the deleterious effects of H_2_O_2_-induced oxidative stress on malaria parasite viability are well established [4], [9], and there is increasing evidence that some antimalarials exert their effect, at least in part, through oxidative mechanisms (e.g. [16], [17]), the mechanisms by which oxidative stress disrupt parasite function are not fully understood. In this study we have shown that the oxidising agent H_2_O_2_, at concentrations comparable to those to which the parasite may be exposed *in vivo* ([35] cited in [4]), causes a decrease in parasite ATP levels and a profound disruption of intracellular pH regulation: an acidification of the parasite cytosol and an alkalinisation of the DV.

Generation of ATP in the intraerythrocytic malaria parasite is thought to be wholly via glycolysis [36], and compounds that inhibit glycolysis [28] or that inhibit glucose uptake into the parasites [37] induce a rapid decrease in parasite [ATP]_i_ [38] and a cessation of parasite growth [28]. Inhibition of glycolysis by H_2_O_2_ has been reported previously in several other cell types [39], [40], [41], [42], [43], and has been shown to be due to the inhibition of the glycolytic enzyme glyceraldehyde-3-phosphate dehydrogenase by H_2_O_2_ [39], [40], [43], [44]. The inhibition of glyceraldehyde-3-phosphate dehydrogenase is attributed to S-thiolation of the enzyme [45]. Inhibition of glycolysis by H_2_O_2_ in other cell-types is also manifest as a decrease in the ATP concentration [41], [43] from which some cells were shown to recover over time [41]. It is likely that the reversible H_2_O_2_-induced decrease in [ATP]_i_ observed in the malaria parasites (Fig. 2B) occurs through a similar inhibition of glyceraldehyde-3-phosphate dehydrogenase, though this is yet to be demonstrated directly. Alternative targets (e.g. other glycolytic enzymes, or the parasite's hexose transporter [46]) cannot be ruled out, and nor is it clear whether there may be an increase in ATP-consumption associated with combating the associated oxidative stress, which might contribute to the H_2_O_2_-induced decline in ATP levels.

The plasma membrane V-type H^+^-ATPase plays a key role in the regulation of cytosolic pH [24], [25], [31], and in generating a large, inwardly negative potential across the plasma membrane [47]. It has been shown previously that depletion of parasite ATP, by removal of glucose from the medium [25], [48], or by inhibition of glucose uptake [38], causes pH_i_ to decrease. The DV V-type H^+^-ATPase plays a key role in maintaining the acidic pH of the DV, and depletion of parasite ATP, by removal of glucose from the medium results in DV alkalinisation [23]. The extent to which the H_2_O_2_-induced decrease in parasite [ATP] might contribute to the observed decrease in pH_i_, and increase in pH_DV_ is not clear. Although a decrease in the parasite's ATP concentration might be expected to slow the rate of H^+^ pumping by the parasite's V-type H^+^-ATPases the [ATP]-dependence of these proteins has not, to our knowledge, been investigated. In previous studies on yeast, plant and bovine V-type H^+^ ATPases, the K_m_ for ATP was found to be 0.2 mM [49], 0.6 mM [50] and 0.15 mM [51], respectively. If the V-type H^+^ ATPase of the malaria parasite has a similar K_m_ then a decrease of [ATP] down to 0.9 mM (on exposure of parasite to 2 mM H_2_O_2_) and to 0.45 mM (on exposure of parasite to 10 mM H_2_O_2_) might be expected to result in a significant decrease in V-type H^+^ ATPase activity. However it should be noted that the previous estimates of the K_m_ were not made in intact cells and the K_m_ of the V-type H^+^ ATPase in an intact malaria parasite, and the extent to which the decreases in [ATP] seen here might have contributed to the loss of pH control, remains an open question.

As was clear from the data obtained here with digitonin-permeabilised parasites (Fig. 2C), quite apart from its effects on cellular ATP levels, H_2_O_2_ caused a direct inhibition of the V-type H^+^-ATPase on the DV, inhibiting its function even when ATP was supplied exogenously. H_2_O_2_, as well as other oxidising agents, has been shown to inhibit the V-type H^+^-ATPase activity in a number of other cell types, including mammalian [52], [53] and plant [54] cells, and the fungus *Neurospora crassa* [55]. Inhibition of the bovine V-type H^+^-ATPase under oxidising conditions is thought to result from the formation of a disulphide bond between Cys-254 and Cys-532 of subunit A of the multimeric protein [56].

By contrast with the DV H^+^-ATPase, the DV H^+^-PPase was refractory to inhibition by H_2_O_2_ (Fig. 4), raising the possibility that the H_2_O_2_-insensitive H^+^-PPase activity might, under some conditions, provide a means for the parasite to maintain the acidity of its DV during periods of exposure to oxidative stress.

Wissing *et al.* have reported previously that light-induced generation of hydroxyl radicals resulted in a pronounced acidification of the parasite cytosol [19]. The acidification was attributed to selective disruption of the DV membrane, the primary evidence for which was the redistribution within the parasite of the lysotropic membrane-permeant dyes acridine orange and Lysosensor blue in response to repeated illumination. Del Pilar Crespo *et al.* [57] reported a similar redistribution of Lysosensor blue fluorescence in response to exposure of cells to the antimalarial artemisinin, for as little as four hours. As illustrated in Fig. 5A and B, exposure of parasites to H_2_O_2_ caused a redistribution of acridine orange fluorescence very similar to that seen in response to laser illumination. However, neither exposure to a high concentration of H_2_O_2_, nor H_2_O_2_-exposure followed by illumination with high light levels resulted in DV membrane disruption. The data presented here do provide an alternative possible explanation for the cytosolic acidification and redistribution of lysotropic dyes observed in the previous studies. In the present study, oxidation-induced inhibition of the plasma membrane and DV membrane V-type H^+^-ATPase resulted in acidification of the cytosol and alkalinisation of the DV, with the result that the pH gradient across the DV membrane was lost. In the absence of such a gradient, membrane-permeant lysotropic dyes would be expected to redistribute throughout the cell. In this scenario it is the inhibition of the plasma membrane and DV H^+^-ATPases that is responsible for the oxidation-induced acidification of the cytosol (as seen by Wissing *et al.* (2002)). Similarly, it is the inhibition of the DV H^+^-ATPase, and the consequent loss of the pH gradient across the DV membrane, rather than a loss of DV membrane integrity, that accounts for the oxidation-induced redistribution of acridine orange and Lysosensor blue within the parasite.

In conclusion we have shown here that oxidative stress, induced by exposure to the oxidising agent H_2_O_2_, results in a fall in parasite ATP, as well as inhibition of the proton-pumping H^+^-ATPase, with a consequent loss of pH control in both the cytosolic and digestive vacuole compartments. By contrast with the H^+^-ATPase, the DV H^+^-PPase is not susceptible to inhibition by H_2_O_2_. The H_2_O_2_-induced fall in ATP is likely to be due to oxidative inhibition of the glycolytic enzyme glyceraldehyde-3-phosphate dehydrogenase, as has been demonstrated to occur in other cell-types. Both glycolysis [28] and the parasite's V-type H^+^-ATPase [58] are of interest as potential antimalarial drug targets, and it is an open question as to whether the inhibition of these pathways and proteins by oxidative stress may play a role in the mechanisms of action of antimalarials currently in use.
